# Precision Medicine Enables More TNM-Like Staging in Patients With Chronic Limb Threatening Ischemia

**DOI:** 10.3389/fcvm.2021.709904

**Published:** 2021-07-16

**Authors:** Katharine L. McGinigle, Nikki L. B. Freeman, William A. Marston, Alik Farber, Michael S. Conte, Michael R. Kosorok, Corey A. Kalbaugh

**Affiliations:** ^1^Department of Surgery, School of Medicine, The University of North Carolina at Chapel Hill, Chapel Hill, NC, United States; ^2^Department of Biostatistics, Gillings School of Global Public Health, The University of North Carolina at Chapel Hill, Chapel Hill, NC, United States; ^3^Department of Surgery, Boston University School of Medicine, Boston, MA, United States; ^4^Department of Surgery, University of California, San Francisco, San Francisco, CA, United States; ^5^Department of Public Health Sciences, Clemson University, Clemson, SC, United States; ^6^Department of Bioengineering, Clemson University, Clemson, SC, United States

**Keywords:** peripheral arterial disease, vascular medicine, amputation free survival, precision medicine, outcomes research, chronic limb threatening ischemia, disease staging system

## Abstract

**Introduction:** In cancer, there are survival-based staging systems and tailored, stage-based treatments. There is little personalized treatment in vascular disease. The 2019 Global Vascular Guidelines on the Management of CLTI proposed successful treatment hinges upon Patient risk, Limb severity, and ANatomic complexity (PLAN). We sought to confirm a three axis approach and define how increasing severity affects mortality, not just limb loss.

**Methods:** Patients revascularized for incident CLTI at our institution from 2013 to 2017 were included. Outcomes were mortality, limb loss, the composite endpoint of amputation-free survival. Using Bayesian machine learning, specifically supervised topic modeling, clusters of patient features associated with mortality were formed after controlling for revascularization type. Patients were assigned to the cluster they belonged to with highest probability; clusters were characterized by analyzing the characteristics of patients within them. Patient outcomes were used to order the clusters into stages with increasing mortality.

**Results:** We defined three distinct clusters as the basis for patient- and limb-centered stages. Across stages, rates of 1-year mortality were 7.6, 13.8, 18.9% and rates of amputation-free survival were 84.8, 79.3, and 63.2%. Stage one had patients with rest pain and previous revascularization who were less likely to have wounds, diabetes, and renal disease. Stage two had doubled mortality, likely related to diabetes prevalence. Stage three is characterized by high rates of complicated comorbidities, particularly end stage renal disease, and significantly higher rate of limb loss (22.6 vs. 8% in stages one and two).

**Conclusion:** Using precision medicine, we have demonstrated clustering of CLTI patients that can be used toward a robust staging system. We provide empiric evidence for PLAN and detail about how changes in each variable affect survival and amputation-free survival.

## Introduction

The Global Vascular Guidelines on the Management of Chronic Limb-threatening Ischemia (CLTI) describes many of the challenges in treating patients with CLTI and reinforces the importance of having strong, evidence based revascularization plans that include medical and surgical therapies (1). These guidelines propose that successful treatment of CLTI hinges upon not just treatment of the limb, but management of the systemic atherosclerotic process. Specifically, there is a proposal to stage patients based on three independent axes: patient risk, limb severity, and anatomic complexity (1). Death in patients with CLTI is rarely caused by their limb-based atherosclerosis or wounds, but is most often due to the atherosclerotic burden in other vascular beds. Risk of myocardial infarction and stoke can be affected (either positively or negatively) by treatments to maximize limb-based outcomes; therefore, staging systems including survival are needed in order to direct the most appropriate limb-based care. While there are cardiac risk calculators and important scoring systems like Wound, Ischemia, Foot Infection (WIFI) (2) and the newly proposed Global Anatomic Staging System (GLASS) (1), in vascular surgery, we do not yet have sufficient data to support staging systems and related treatment protocols tailored to each individual patient's type or severity of peripheral arterial disease.

The range of PAD patients can be likened to cancer patients. For example, a patient in rest pain (small tumor) with focal femoral artery occlusion (no nodal spread) without other end-organ problems (no metastasis) is similar to a patient with stage 1 or 2 cancer. It is likely that treatments will be less complex and there will be good chances of limb preservation and long-term survival. On the other hand, patients with an ischemic wound (large tumor), multi-focal arterial occlusions (nodal involvement) and renal failure (metastatic disease) are likely to have poor survival rates, similar to stage 4 cancer, and a focus on palliation and end of life decision making should play a larger role in the treatment choices even if limb revascularization is pursued. Extensive clinical research in the cancer field has resulted in staging systems for every type of cancer, followed with tailored, specific stage-based treatments based on these outcome predictors.

In addition to overall systemic disease severity, it is reasonable that limb severity measured by WIFI and anatomic complexity measured by GLASS, contribute to outcomes. However, it is unknown to what degree each of these measures or combinations of measure contribute nor how they contribute *via* interaction with underlying patient risk. Treatment decisions have impacts on limb preservation and mortality, but clinicians are ill-equipped to guide patients through these decisions as there is little data on what outcomes are achievable and at what cost (financially, socially, etc.). A staging system centered on survival is needed to guide a multi-disciplinary approach to achieve patient-centered, in addition to limb-centered, treatment decisions.

The purpose of our study was to pilot the precision medicine methods needed to expand upon and improve existing CLTI mortality and limb loss models (2, 3) thereby allowing for more fully delineated CLTI staging. The secondary aim was to provide empirical evidence for a three axis approach to staging proposed in the most recent vascular guidelines and to define how increasing severity in each axis affects limb loss and mortality. This will allow for continued investigation in larger, multi-center cohorts and also for thoughtful discussions related to appropriateness of treatment choices in subsets of the CLTI patient population.

## Materials and Methods

### Study Population

Our cohort included consecutive patients that met hemodynamic and symptomatic criteria for CLTI who underwent open or endovascular revascularization for CLTI.at our institution between April 2013 and October 2017.

### CLTI Definition

To be diagnosed with CLTI, patients had to meet both hemodynamic and symptomatic criteria. Hemodynamic criteria were defined as an ankle-brachial index <0.50, ankle pressure <70 mmHg or a toe pressure <50 mmHg (4). We obtained PVL data using the Syngo (Siemens Medical Solutions USA) software program that contains hemodynamic information such as brachial pressures, ankle pressures, toe pressures, ankle-brachial index (ABI) measurements, and toe-brachial index (TBI) measurements. Symptoms of CLTI included ischemic rest pain, ischemic ulceration or ischemic gangrene of the lower extremities, identified by billing codes (5) and record abstraction (done both manually and with natural language processing).

### Demographics and Comorbidities

We measured patient age at the time of incident revascularization procedure, as well as their race and sex. For parsimony in this pilot, comorbidities were coded as a simple absent vs. present, or in some cases absent vs. present vs. present in an advanced/complicated form. Comorbidities included hypertension (uncomplicated, complicated), diabetes mellitus (uncomplicated, complicated), coronary artery disease, congestive heart failure, renal disease (chronic kidney disease, end-stage renal disease), smoking, chronic obstructive pulmonary disease, and hypercoagulability. Uncomplicated hypertension was defined by ICD-10 codes for essential hypertension; whereas, complicated hypertension included ICD-10 codes for malignant hypertensive disease and hypertensive disease with sequelae like heart failure or kidney disease. Similarly, complicated diabetes included all of the codes for diabetes with other specified manifestations such as retinopathy, nephropathy, and neuropathy.

### Limb Severity and Anatomic Complexity

Each patient received retrospectively determined WIfI ischemia grades based on degree of ischemia from the index peripheral vascular lab test. The best estimates of wound severity were based off diagnostic and procedure codes. For example, codes for digit amputation and debridement of skin and subcutaneous tissue mapped to a WIfI wound grade 1 (minor tissue loss); transmetatarsal amputation or debridement of muscle and bone mapped to a WIfI wound grade 2 (major tissue loss); codes for non-traditional foot amputations and calcanectomy mapped to WIfI wound grade 3 (extensive tissue loss). Neither chart review nor diagnostic codes were precise enough to reliably include the infection grade, so this was simply classified as presence or absence of foot infection. Natural language processing was used to evaluate arteriogram reports for stenotic and occluded vessels. To ensure accuracy of the collected anatomic data, these data were compared to the arterial duplex reports and if there was any discrepancy, manual review of the arteriogram was performed. The diseased vessels were categorized as inflow (iliac artery), outflow (femoropopliteal arteries), runoff (infrapopliteal arteries), and multi-segment arterial disease. More precise categorization of anatomic complexity was not possible as TASC classifications were not available in the EHR and there is no current validated tool in use to address the range of disease in the infrapopliteal arteries.

### Outcomes

The primary outcome is mortality. Mortality data was obtained *via* the Carolina Data Warehouse (CDW) (6) and the state death record. Secondary outcomes, also chosen a priori, are limb amputation and amputation-free survival. Limb amputation was identified by procedure codes in the CDW. Failure of amputation free survival was determined by the first event of death or limb loss.

### Statistical Analysis

Demographic, comorbid, and CLTI-related variables for the cohort studied were described using the median and IQR for continuous variables and counts and percentages for categorical and binary variables. Descriptions were reported for the whole cohort and separately by the type of revascularization procedure received. In accordance with the STROBE statement for reporting outcomes from observational studies, we do not include *p*-value comparisons between the two groups in our description (7).

Clusters were formed using a supervised latent Dirichlet allocation (sLDA) topic model and posterior samples were obtained *via* Gibbs sampling (8, 9). sLDA is a topic modeling method from the natural language processing literature that models the words in the corpus of documents and discovers latent topics within those documents; unlike many clustering methods, sLDA uses “supervision” to guide cluster formation so that clusters are predictive of the desired response. We considered our sample as a corpus of documents, each patient-limb diagnosed with CLTI as a document, and patient and patient-limb baseline features as words to be clustered into topics. Topic formation allows for words to belong to multiple topics (e.g., “bank” may refer to a river bank or to a financial institution); in its application to CLTI staging, our clusters are characterized by groups of concurrent patient baseline features and those features are not limited to one particular cluster. For example, one distinct cluster may be identified by patients with diabetes and hypertension, and another might be identified by patients with diabetes and end-stage renal disease, capturing the complexity of the CLTI patient population without binary classification based on comorbid status (e.g., all diabetic patients belonging to one single cluster).

Because our cohort is retrospective and revascularization strategies were not randomly assigned, a modification was made the standard sLDA model to include a model for the probability of treatment (open or endovascular revascularization) conditional on baseline patient characteristics (propensity score) and to incorporate that information to cluster formation. Our binary outcomes were used to provide supervision and entered the model with a probit likelihood. The data were analyzed using 2, 3, 4, and 5 clusters. The number of clusters in the final model was 3 since there was a clear point of diminishing returns for adding more clusters beyond three (**Figure 2**). Clinician examination of the patient features associated with each cluster confirmed a practical clinical applicability. After cluster formation, the probabilities of patients belonging to a particular cluster were also computed; patients were assigned to the cluster to which they belonged to with the highest probability. Clusters were characterized by the features with which they were most often associated and further characterized by analyzing the characteristics of the patients within them.

For this hypothesis-generating pilot study, we emphasize that our model describes our cohort and provides insight on the feasibility of delineating limb-based and survival-based stages of CLTI. Based on guidance from the American Statistical Association, we do not conduct null hypothesis significance tests to compare the latent stages discovered as one might would in a formal confirmatory study (10–12). Further, we do not consider how treatment or complications may affect outcomes as the purpose of this analysis is to define stages at the time of diagnosis to then assist with shared decision making around the most appropriate treatment choices given risk of limb loss and death based on presenting patient characteristics.

Analyses were performed using R version 3.6.0 and the Rcpp package version 1.0.3. This study, in which no informed consent was required, was approved by the Institutional Review Board at the University of North Carolina.

## Results

The final cohort included 285 patients with CLTI; 197 underwent an endovascular revascularization and 88 had an infrainguinal bypass operation (Table 1). Of note, there were also 287 patients with CLTI who did not undergo revascularization during this same time period, and they were excluded from this study. Of the excluded patients, 220 were initially treated with wound care alone, 31 underwent primary major limb amputation, and 36 did not have any followup. Patients included in the cohort had a median age of 64 years (IQR: 15). The most common comorbidities present overall included: uncomplicated hypertension (90%), positive smoking history (75%), hyperlipidemia (72%), and uncomplicated diabetes (60%). In general, patients in the endovascular cohort were slightly more comorbid than the open surgery group. Patients in the endovascular cohort had a higher prevalence of dementia (8 vs. 2%), complicated diabetes (56 vs. 48%), complicated hypertension (44 vs. 32%), and chronic kidney disease (37 vs. 26%) including nearly double the proportion of patients affected by ESRD (17 vs. 9%).

**Table 1:** Demographic and comorbidities of overall population.

	**Overall cohort** ** (*N* = 285)**	**Open** ** (*N* = 88)**	**Endovascular** ** (*N* = 197)**
Age, median (IQR)	64.2 years (15.6)	63.6 years (13.9)	64.8 years (17.2)
Sex, male (%)	169 (59.3%)	52 (59.1%)	117 (59.4%)
Race/ethnicity, *n* (%)			
Non-Hispanic White	150 (52.6%)	51 (58.0%)	99 (50.3%)
Non-Hispanic Black	98 (34.4%)	26 (29.5%)	72 (36.5%)
Hispanic	17 (6.0%)	3 (3.4%)	14 (7.1%)
Comorbidities, *n* (%)			
Anemia	128 (44.9%)	40 (45.5%)	88 (44.7%)
Cerebrovascular disease	101 (35.4%)	33 (37.5%)	68 (34.5%)
Chronic pulmonary disease	91 (31.9%)	30 (34.1%)	61 (31.0%)
Coagulopathy	54 (18.9%)	21 (23.9%)	33 (16.8%)
Congestive heart failure—complicated	37 (13.0%)	8 (9.1%)	29 (14.7%)
Congestive heart failure	94 (33.0%)	26 (29.5%)	68 (34.5%)
Coronary artery disease	163 (57.2%)	54 (61.4%)	109 (55.3%)
Dementia	17 (6.0%)	2 (2.3%)	15 (7.6%)
Diabetes—complicated	152 (53.3%)	42 (47.7%)	110 (55.8%)
Diabetes	74 (26.0%)	20 (22.7%)	54 (27.4%)
Diabetes—uncomplicated	171 (60.0%)	49 (55.7%)	122 (61.9%)
Hyperlipidemia	205 (71.9%)	67 (76.1%)	138 (70.1%)
Hypertension—complicated	114 (40.0%)	28 (31.8%)	86 (43.7%)
Hypertension—uncomplicated	258 (90.5%)	80 (90.9%)	178 (90.4%)
Myocardial infarction	89 (31.2%)	27 (30.7%)	62 (31.5%)
Obesity	55 (19.3%)	18 (20.5%)	37 (18.8%)
Renal disease—CKD	87 (30.5%)	23 (26.1%)	64 (32.5%)
Renal disease—complicated	95 (33.3%)	23 (26.1%)	72 (36.5%)
Renal disease—ESRD	42 (14.7%)	8 (9.1%)	34 (17.3%)
Renal disease	42 (14.7%)	6 (6.8%)	36 (18.3%)
Smoking	216 (75.8%)	75 (85.2%)	141 (71.6%)
Venous insufficiency	38 (13.3%)	14 (15.9%)	24 (12.2%)
Weight loss	51 (17.9%)	17 (19.3%)	34 (17.3%)
Venous thromboembolism	85 (29.8%)	30 (34.1%)	55 (27.9%)
WIfI characteristics			
Wound class 0	89 (31.2%)	27 (30.7%)	62 (31.5%)
Wound class 1	69 (24.2%)	20 (22.7%)	49 (24.9%)
Wound class 2	59 (20.7%)	19 (21.6%)	40 (20.3%)
Wound class 3	68 (23.9%)	22 (25.0%)	46 (23.4%)
Ischemia class 3	249 (87.4%)	82 (93.2%)	167 (84.8%)
Anatomy			
Inflow (iliac) disease	194 (68.1%)	63 (71.6%)	131 (66.5%)
Outflow (femoropoliteal) disease	202 (70.9%)	68 (77.3%)	134 (68.0%)
Runoff (infrapopliteal) disease	174 (61.1%)	41 (46.6%)	133 (67.5%)
Multilevel disease	220 (77.2%)	74 (84.1%)	146 (74.1%)

### Differences in Traits by Stages of CLTI

**Figure 1:**
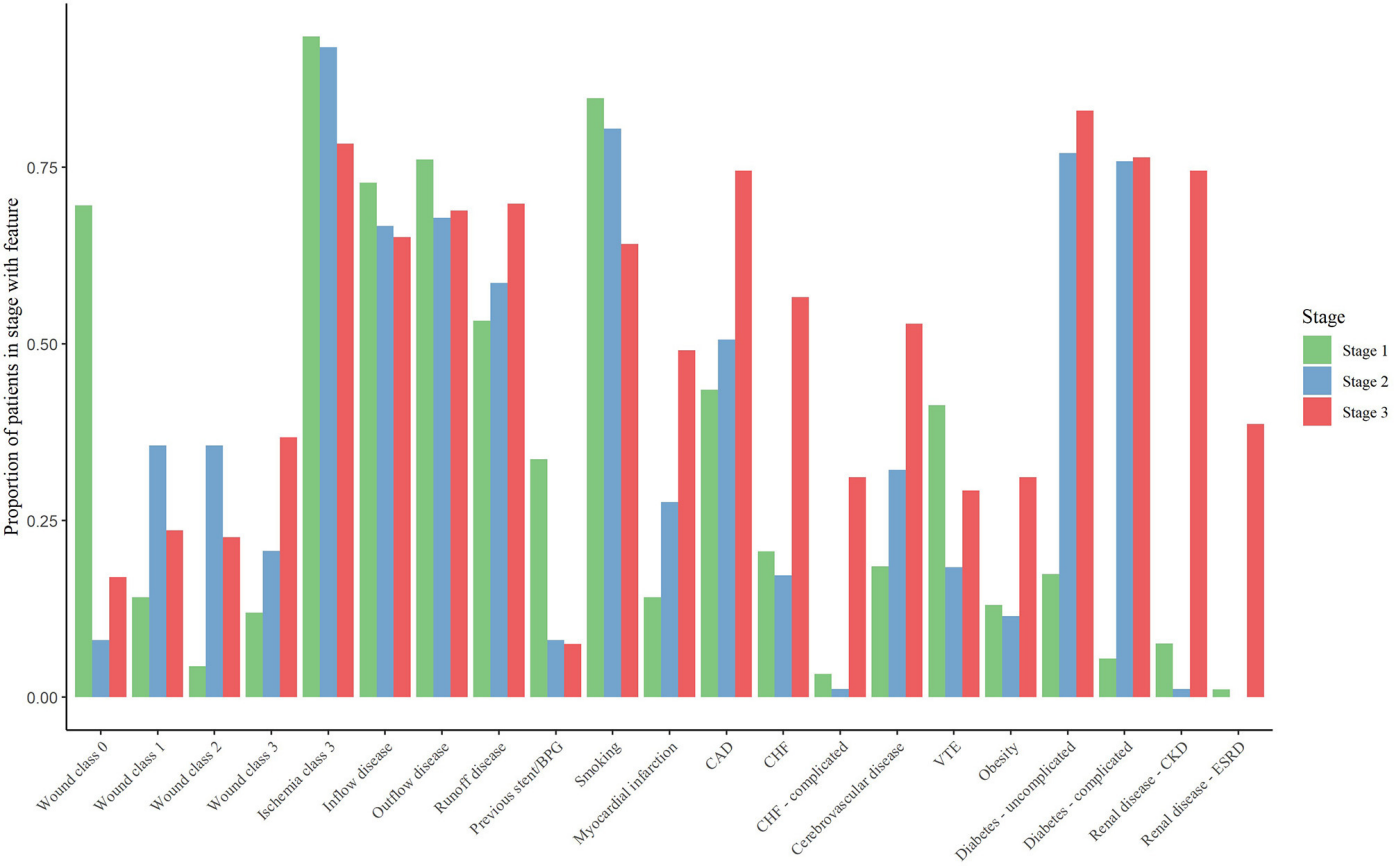
Characteristics of patients within each stage.

**Figure 2:**
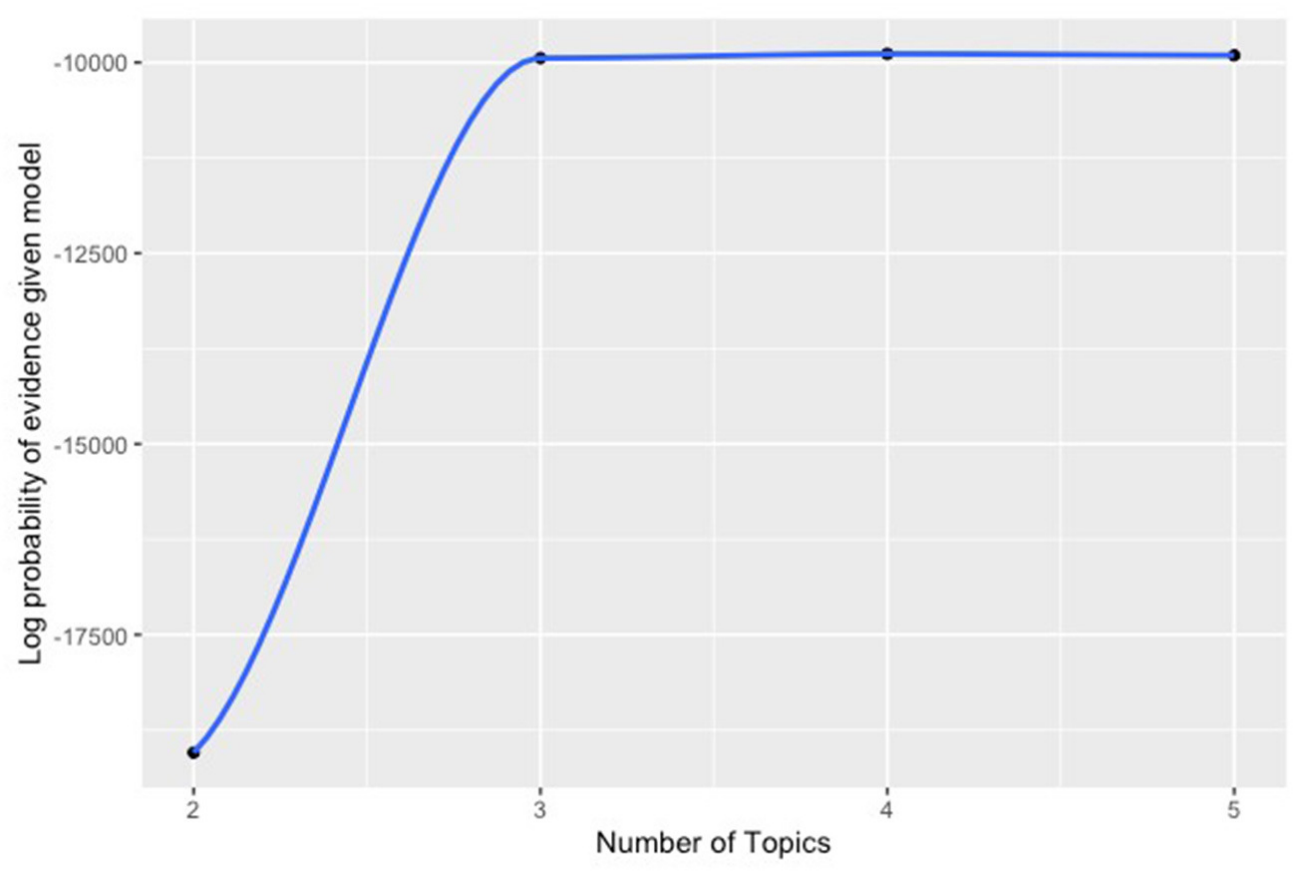
Log posterior probability of the model given the evidence.

We delineated three distinct clusters within the CLTI cohort that can be used as the basis for patient- rather than limb-centered stages (Table 2 and Figures 1, 2). The outcomes associated with each cluster was assessed and the stages were defined so that Stage 1 is the cluster with the lowest mortality and Stage 3 the cluster with the highest mortality.

**Table 2:** Characteristics of CLTI patients belonging to each stage.

	**Stage 1** **(*N* = 92)**	**Stage 2** **(*N* = 87)**	**Stage 3** ** (*N* = 106)**
Procedure			
Endovascular	61 (66.3%)	55 (63.2%)	81 (76.4%)
Open	31 (33.7%)	32 (36.8%)	25 (23.6%)
Comorbidity			
Anemia	26 (28.3%)	28 (32.2%)	74 (69.8%)
Cerebrovascular disease	17 (18.5%)	28 (32.2%)	56 (52.8%)
Chronic pulmonary disease	43 (46.7%)	16 (18.4%)	32 (30.2%)
Coagulopathy	26 (28.3%)	5 (5.7%)	23 (21.7%)
Congestive heart failure—complicated	3 (3.3%)	1 (1.1%)	33 (31.1%)
Congestive heart failure	19 (20.7%)	15 (17.2%)	60 (56.6%)
Coronary artery disease	40 (43.5%)	44 (50.6%)	79 (74.5%)
Dementia	1 (1.1 %)	7 (8.0%)	9 (8.5%)
Diabetes—complicated	5 (5.4%)	66 (75.9%)	81 (76.4%)
Diabetes	2 (2.2%)	28 (32.2%)	44 (41.5%)
Diabetes—uncomplicated	16 (17.4%)	67 (77.0%)	88 (83.0%)
Hyperlipidemia	52 (56.5%)	67 (77.0%)	86 (81.1%)
Hypertension—complicated	10 (10.9%)	1 (1.1%)	103 (97.2%)
Hypertension—uncomplicated	73 (79.3%)	80 (92.0%)	105 (99.1%)
Myocardial infarction	13 (14.1%)	24 (27.6%)	52 (49.1%)
Obesity	12 (13.0%)	10 (11.5%)	33 (31.1%)
Renal disease—CKD	7 (7.6%)	1 (1.1%)	79 (74.5%)
Renal disease—complicated	5 (5.4%)	0 (0.0%)	90 (84.9%)
Renal disease—ESRD	1 (1.1%)	0 (0.0%)	41 (38.7%)
Renal disease	0 (0.0%)	0 (0.0%)	42 (39.6%)
Smoking	78 (84.8%)	70 (80.5%)	68 (64.2%)
WIfI			
Wound class 0	64 (69.6%)	7 (8.0%)	18 (17.0%)
Wound class 1	13 (14.1%)	31 (35.6%)	25 (23.6%)
Wound class 2	4 (4.3%)	31 (35.6%)	24 (22.6%)
Wound class 3	11 (12.0%)	18 (20.7%)	39 (36.8%)
Ischemia class 3	86 (93.5%)	80 (92.0%)	83 (78.3%)
Anatomy			
Inflow disease	67 (72.8%)	58 (66.7%)	69 (65.1%)
Outflow disease	70 (76.1%)	59 (67.8%)	73 (68.9%)
Runoff disease	49 (53.3%)	51 (58.6%)	74 (69.8%)

Stage I included 92 patients and was characterized by patients with previous revascularization attempts. Compared to stage II and stage III patients, stage I patients were the least comorbid but did have a higher prevalence of smoking (85%), COPD (47%), venous thromboembolism (41%), and venous insufficiency (22%). However, stage I patients were generally found to have low prevalence of cardiovascular conditions caused by these risk factors. These patients were most likely to have exclusive inflow disease (73%).

We identified 87 patients as having stage II disease. Compared to stage I patients, those with stage II disease had slightly higher prevalence of several comorbidities including a much higher prevalence of complicated diabetes (76 vs. 5%) and cerebrovascular disease (32 vs. 19%). Renal disease was observed in only one person with stage II disease (1%).

Stage III disease included 106 patients characterized by high rates of complicated comorbidities and end-stage processes of various cardiovascular diseases. At least three-fourths of stage III patients had complicated diabetes (76%), coronary artery disease (75%), hypertension (97%), or renal disease (85%). More than half of patients had cerebrovascular disease (53%) or congestive heart failure (57%). Stage III patients were most likely to have runoff disease (70%). The presence of severe ischemia was common across all stages and was not a delineating factor between the stages.

### Outcomes by Stages of CLTI

Across the three stages, 1-year mortality rates were 7.6, 13.8, and 18.9%, respectively (Table 3). Limb loss rates at 1 year were 8.7, 8.0, and 22.6% across the three stages. One-year amputation-free survival was 84.8% in stage I patients, 79.3% in stage II patients and 63.2% in stage III patients. While stage I and stage II patients had similar limb loss rates, mortality was almost double in stage II patients as compared to stage I patients. Stage III patients had a 2.5 times higher rates of limb loss than both stage I and stage II patients. Stage III patients had a more than double mortality rate than stage I patients.

**Table 3:** Outcomes by stage.

**Stage**	**1-year mortality Percent (Nominal 95% CI)**	**1-year limb loss Percent (Nominal 95% CI)**	**1-year amputation-free survival Percent (Nominal 95% CI)**
Stage 1 (*n* = 92)	7.6% (2.2, 13.0%)	8.7% (2.9, 14.5%)	84.8% (77.5, 92.1%)
Stage 2 (*n* = 87)	13.8% (6.6, 21.0%)	8.0% (2.3, 13.7%)	79.3% (70.8, 87.8%)
Stage 3 (*n* = 106)	18.9% (11.4, 26.4%)	22.6% (14.6, 30.6%)	63.2% (54.0, 72.4%)

## Discussion

Our study incorporated information on patient demographics and comorbid conditions, as well as the WIfI score and anatomic extent of disease, to delineate different cohorts of patients with CLTI at the time of diagnosis. We successfully demonstrated clustering of CLTI patients into three relatively distinct stages with each stage representing differing traits and increasing mortality following revascularization. Specifically, patients with Stage I CLTI (the least severe category) had much lower than average reported mortality rates and very good amputation free survival, so everything should be done to assure that these patients get optimal medical therapy and the most durable revascularization. Patients with Stage III CLTI (the most severe category) were characterized by high rates of complicated comorbidities, particularly renal disease, and <2/3 of these patients survived for 1 year with an intact limb, which may indicate that less invasive, or even more palliative interventions, are better suited to this sub-population. The majority of the total cohort had severe ischemia, but presence of severe ischemia did not appear to be an independent factor between the stages. Our findings indicate studies on larger cohorts to solidify the role of combined survival-based, and limb-based, treatment strategies is warranted.

Marked population-level increases in important risk factors such as diabetes and chronic kidney disease are changing the management of patients with vascular diseases and sequelae such as CLTI (13, 14). Assessment of patient risk in CLTI patients is a critical component of an appropriate management strategy that is challenged by these twenty-first century shifts in comorbid conditions. The VQI cardiac risk calculator can help predict major adverse cardiac event in the post-operative period following infrainguinal bypass, but does not apply to patients undergoing endovascular therapy, and cannot predict cardiac risk outside of the immediate post-operative period (3). We found that Stage III patients were most likely to have a series of complicated comorbidities, including renal disease, diabetes, and hypertension. Patients with these series of comorbidities likely have disease in all three major vascular beds and represent the group with the highest 1-year mortality rates and the highest limb loss rates. Medical optimization of patients with CLTI remains understudied; however, it has been shown that improved adherence to high-dose statin therapy improves survival in patients with PAD (15). Our findings suggest that aggressive management of the conditions that cause severe PAD sequelae must be prioritized, perhaps even before surgical intervention is offered, but further prospective precision medicine related to best sequences of care is needed.

The SVS WIfI score is a consensus-derived and validated classification system used to stage the *limb(s)* axis component of the three-axis approach recommended by GVG in patients with CLTI (1, 2). WIfI includes severity grading of wounds, ischemia, and foot infections and has repeatedly been shown to predict amputation risk (16, 17). Interestingly, in our study we found that the degree of ischemia did not drive the differences in *survival* based stage. Instead the increasing wound severity at presentation was clearly a differentiating factor between survival based stages. In stage I, most of the cohort presented with rest pain and no wounds, and the majority of the wound grade 3 (full thickness forefoot or hindfoot ulcer) patients were clustered in stage III. While WIfI was not designed to predict survival or amputation free survival, it remains an important factor in predicting limb outcomes and should be considered in any staging system.

The anatomic complexity of the stenotic or occluded arteries is related to the technical success of the revascularization and the patency rates. In our study, the presence of multi-level arterial disease mattered less than expected for our primary outcome, mortality. We did find that patients in stage III were much more likely to have tibial arterial disease, but this is a pattern that matches with the comorbid conditions of diabetes and ESRD, which are also frequent in this stage, so anatomy is unlikely to be an independent risk factor. The newly proposed GLASS specifically characterizes infrapopliteal arteries, and it may be that certain detailed combinations of arterial occlusions are more predictive of amputation, and even death. Unfortunately, GLASS is not validated or in frequent use, but future study and further delineation of severity of tibial disease will be important and may show a separation between stage II and III.

Comparisons of different revascularization techniques based on anatomic lesions are rare and there is little standardization of practice (18). Although we hope the upcoming results of the BEST-CLI trial and validation studies of GLASS will provide guidance, the current literature is primarily focused on short-term bypass or stent patency outcomes. In the case of CLTI patients with high expected cardiovascular mortality, many of these patients will not survive to realize the benefit of revascularization, and it may be that wound care alone is appropriate treatment (19–22). Clinically, it is not surprising that the patients with the most advanced system-based and limb-based atherosclerosis have poor surgical outcomes, but this begs the question of whether standard surgical and endovascular approaches are the best treatment choice. Regardless of the cause of death and whether it is directly linked to CLTI treatment, most patients will decide against invasive procedures or multiple hospitalizations, especially at the end of life, if they know that this type of aggressive care is futile (23). The cohort of patients with CLTI treated with wound care without revascularization were not included in this study due to concerns related to significant selection bias in a retrospective study, but will be important to consider in future prospective work as it will help further delineate best care for these most fragile patients. Nonetheless, our findings are an important step toward being able to have evidence-based conversations about interventional management, including palliative options, with our patients.

The information in this pilot study can be used toward development of a data-driven robust staging system- similar to cancer- that allows vascular specialists to better communicate with patients about expected outcomes and appropriate treatment choices at the time of the CLTI diagnosis. As with all pathologies, treatment response, complications, and worsening comorbidities will contribute to outcomes and are important to consider over the longitudinal care of a CLTI patient, but this research focuses on the presenting characteristics and how they may influence initial treatment decisions.

Our study has several limitations that should be acknowledged. First, this is a retrospective study and treatments were not randomly assigned. Our data do not imply causality and are meant to serve as pilot information for a larger trial. Second, data was abstracted from our electronic health record and is subject to the limitations of EHR data, and this includes not being able to accurately include foot infection scores into our WIfI estimations. Third, our data originate from a single institution and we acknowledge that our practice patterns and patient population may not represent the broader community of vascular surgeons treating patients with CLTI.

Our study has several strengths that are important to discuss. Our use of machine learning methods and natural language processing represent state-of-the-art methodological advances over previous literature. Using these methods, we are able to provide empirical evidence to the consensus derived PLAN framework for managing CLTI. Finally, this project provides clear scientific evidence to expert consensus.

In the future, it will be important to validate this proposed staging system in larger, more generalizable cohorts or even in pooled clinical trial cohorts. Larger patient populations with prospectively collected, detailed clinical and anatomic data will allow for refined clustering and will help define the size of the increments that change a patient's position on each of the three axes in the PLAN framework. At that point, clinical applications like the TNM staging system can be developed for use at the bedside when a CLTI diagnosis is made and treatments are offered.

In conclusion, using precision medicine, we have demonstrated clustering of CLTI patients that can be used toward development of a robust staging system. Our results parallel the axes proposed in PLAN, but provide more detail about how changes in each variable effect survival and amputation free survival after revascularization. Developing a staging strategy that helps clinicians guide patients in which treatment option is likely to provide the best outcome, including chance of survival, will greatly enhance the patient and clinician experience in the management of this very difficult disease.

## Data Availability Statement

The raw data supporting the conclusions of this article will be made available by the authors, without undue reservation.

## Ethics Statement

The studies involving human participants were reviewed and approved by University of North Carolina at Chapel Hill Institutional Review Board. Written informed consent for participation was not required for this study in accordance with the national legislation and the institutional requirements.

## Author Contributions

KM and CK conceived and designed the study. NF organized the database. NF and MK performed the statistical analysis. KM, NF, and CK wrote the first draft of the manuscript. All authors contributed to the manuscript revision, throughout multiple rounds of revision, read, and approved this version of the submitted manuscript.

## Conflict of Interest

The authors declare that the research was conducted in the absence of any commercial or financial relationships that could be construed as a potential conflict of interest. The reviewer MT declared a past collaboration with one of the authors MC to the handling Editor.
